# Micromagnetic Simulations of Submicron Vortex Structures for the Detection of Superparamagnetic Labels

**DOI:** 10.3390/s20205819

**Published:** 2020-10-15

**Authors:** Lukas Wetterau, Claas Abert, Dieter Suess, Manfred Albrecht, Bernd Witzigmann

**Affiliations:** 1 Computational Electronics and Photonics and CINSaT, University of Kassel, 34121 Kassel, Germany; bernd.witzigmann@uni-kassel.de; 2 Physics of Functional Materials, University of Vienna, 1090 Vienna, Austria; claas.abert@univie.ac.at (C.A.); dieter.suess@univie.ac.at (D.S.); 3 Institute of Physics, University of Augsburg, 86159 Augsburg, Germany; manfred.albrecht@physik.uni-augsburg.de

**Keywords:** micromagnetic simulations, superparamagnetic labels, vortex structures, giant magnetoresistance, lab-on-a-chip device

## Abstract

We present a numerical investigation on the detection of superparamagnetic labels using a giant magnetoresistance (GMR) vortex structure. For this purpose, the Landau–Lifshitz–Gilbert equation was solved numerically applying an external z-field for the activation of the superparamagnetic label. Initially, the free layer’s magnetization change due to the stray field of the label is simulated. The electric response of the GMR sensor is calculated by applying a self-consistent spin-diffusion model to the precomputed magnetization configurations. It is shown that the soft-magnetic free layer reacts on the stray field of the label by shifting the magnetic vortex orthogonally to the shift direction of the label. As a consequence, the electric potential of the GMR sensor changes significantly for label shifts parallel or antiparallel to the pinning of the fixed layer. Depending on the label size and its distance to the sensor, the GMR sensor responds, changing the electric potential from 26.6 mV to 28.3 mV.

## 1. Introduction

Magnetoresistive sensors are part of many technical applications and are already implemented in several industrial sectors. Due to their high sensitivity and integration capability, magnetoresistive sensors are also a promising technology in the field of flexible electronics [1], human–computer interactions [2] and biomedicine [3]. In the case of biomedical applications, the detection of magnetic signals can be challenging since human organs generate very small magnetic fields ranging from nT to fT [4]. Therefore, magnetoresistive sensors can be combined with bio-functionalized labels to enhance magnetic interaction. Excited by an external field, these labels generate magnetic stray fields, which can be detected electronically by a magnetoresistive sensor element [5].

In contrast with other technologies, this provides a multitude of advantages. Compared to the detection of fluorescence signals induced by light absorption of biological samples, no interference appears with most biological samples when using magnetic stray fields [5]. Additionally, the strong coupling of the magnetic stray field in magnetically labelled samples with an external magnet offers the opportunity for efficient label separation and transportation [6]. Since magnetic field sensors are already implemented in commercial motion-control applications, CMOS techniques have been developed, which are compatible with low-cost magnetoresistive sensors. This platform design and the analytical techniques can be applied to the development of a biosensor as well [5]. It becomes apparent that a magnetic sensing concept based on magnetoresistive effects is a promising approach for the development of a biomolecule detecting and quantifying sensor [4,5,7,8].

**Figure 1:**
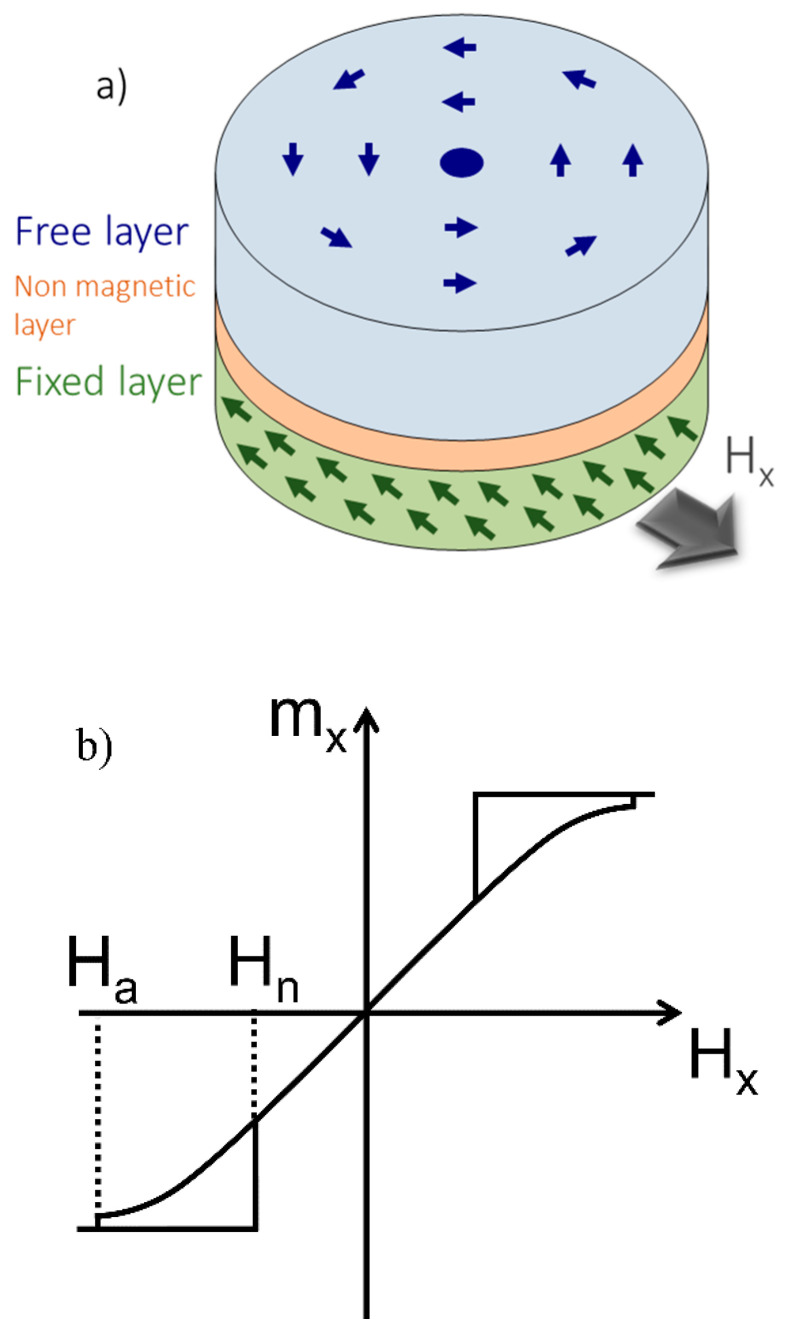
Magnetic vortex characteristics. (**a**) Flux closure state of the magnetic vortex within the free layer of an employed giant magnetoresistance (GMR) stack. (**b**) Characteristic magnetic hysteresis loop of a magnetic vortex with an annihilation field H_a_ and a nucleation field H_n_.

For the detection and quantification of biomolecules, a hysteresis-free response and a high-signal-to-noise ratio are crucial features [9]. In this regard, giant magnetoresistance (GMR) spin-valve structures, including a magnetic vortex free layer (Figure 1a), were introduced, offering a linear sensor response with low magnetic noise [10,11].

Magnetic vortices arise in micron-sized, nanometer-thick, circular ferromagnetic disks, where the lateral extension is much larger than the thickness of the layer. In the absence of a magnetic field, the magnetization curls up in-plane along the edges to minimize magnetostatic energy, leading to a flux closure state with an out of plane magnetization in the center of the disk (vortex core) [12]. In the presence of an external magnetic field, the parallel and antiparallel oriented magnetic domains increase and decrease equally, and the magnetic vortex moves proportionally to the external magnetic field until it annihilates at the annihilation field H_a_ (Figure 1b). This change in the layer’s magnetization results in a change in electrical resistance if the vortex layer represents the free layer of a GMR spin-valve stack [10].

In principle, ferromagnetic and superparamagnetic labels can generate detectable magnetic fields. As the size of several important biomarkers is assigned to the nanometer range, the size of magnetic labels to be detected should be comparable [13]. In this range, most labels show superparamagnetic properties reaching critical diameters smaller than 100 nm [14]. In contrast to superparamagnetic labels, ferromagnetic labels exhibit a nonvanishing magnetization, even in the absence of a magnetic field. As a consequence, ferromagnetic labels are attracted to each other and tend to aggregate. This limits the performance of a sensor and leads to potential health issues due to embolism if an in vivo sensing concept is contemplated [13]. By using superparamagnetic labels, this can be avoided. Superparamagnetic properties arise in small ferromagnetic particles below a critical size that depends on the material properties. In this case, labels will only provide a magnetic moment if a small external field is applied. In the absence of the external field and above the blocking temperature, the spins of the label flip randomly, and the averaged magnetic moment disappears [14]. Hence, the magnetic moment of a superparamagnetic label can be activated by the time the label reaches the detector using an external field (magnetizing field) perpendicular to the sensor surface [15].

In developing a sensor system for the detection of a single label, micromagnetic simulations help understand the interaction of the sensor and the label. Here, the 3D finite element magnum.fe tool with the self-consistent feature as published in [16] is used to introduce a spherical label directly to the simulation domain and vary its diameter, distance, horizontal and vertical position to the surface of a simplified GMR element. By solving the Landau–Lifshitz–Gilbert equation numerically, the changes in the sensor’s magnetization generated by the stray field of the label are determined and used to simulate the GMR response in a post-processing procedure via the self-consistent micromagnetic model [16].

## 2. Simulation Methods and Parameters

Basically, a GMR spin-valve system is fabricated as an array including several hundreds of single GMR elements. In a basic setup, each element consists of a magnetic free layer and a magnetic fixed layer separated by a non-magnetic conducting layer (Figure 1a). The fixed layer is usually designed to have a stable and homogeneous magnetization configuration, which is typically achieved by an antiferromagnetic exchange bias layer [17]. On the other hand, the free layer magnetization is sensitive to changes in the external field as introduced by the magnetic labels. The relative change in fixed and free layer magnetization is reflected in its GMR signal.

### 2.1. Simulation Model

In order to model the free layer’s magnetization rotation due to the label’s stray field, the Landau–Lifshitz–Gilbert equation describes the magnetization dynamics of a three-dimensional magnetic domain [18]:

(1) $$\frac{\partial m}{\partial t}=-\gamma \left(m\;\times \;h_{\mathrm{eff}}\right)\;+\;\alpha \left(m\;\times \;\frac{\partial m}{\partial t}\right)$$

It connects the precessional damped motion of the normalized magnetization m to an effective magnetic field **h**_eff_, where γ and α are the gyromagnetic ratio and the damping constant, respectively. According to the problem setting, the effective field term **h**_eff_ includes the demagnetization field **h**_demag_ and the exchange field **h**_ex_ describing the dipole–dipole interaction and the quantum-mechanical effect of the exchange interaction. In order to simulate the activation of the superparamagnetic label, the effective field is completed by a Zeeman-field contribution **h**_zeeman_ [19].

(2) $$h_{\mathrm{eff}}\;=\;h_{\mathrm{demag}}+\;h_{\mathrm{ex}}+h_{\mathrm{zeeman}}$$

For the numerical solution of the Landau–Lifshitz–Gilbert equation, the 3D finite element method tool magnum.fe is used [20]. The terms of Equation (2) are defined as reported in [20] as well as the corresponding boundary conditions. During the generation of the finite element mesh, the label is placed close to the surface of the soft-magnetic free layer to determine how the layer’s magnetization is affected by the stray field of the label. Subsequently, this free-layer configuration is used to calculate the GMR response of the sensor by solving the self-consistent spin-diffusion model.

Within the spin-diffusion approach, the spin accumulation s is introduced characterizing the excess of one spin orientation due to the magnetization direction of a ferromagnetic material. Its diffusion through the layer stack is defined by the equation of motion in [16], which provokes the spin current **j**_s_. The spin accumulation s is assumed to be always in equilibrium, since it undergoes temporal changes two orders of magnitude smaller compared to the dynamics of the magnetization, which leads to Equation (3) [16].

(3) $$\nabla \;\cdot \;j_{S}+\frac{s}{\tau_{\mathrm{sf}}}+J\frac{s\;\times \;m}{\hbar }=0$$

The spin current **j**_s_ is defined by Equation (4) coupled to the charge current **j**_e_ in Equation (5) via the electric potential u using E = −∇u.

(4) $$j_{s}\;=\;2\beta C_{0}\frac{\mu_{B}}{e}m⊗E\;-\;2D_{0}\nabla s$$

(5) $$j_{e}\;=\;2C_{0}E\;-\;2\beta ’D_{0}\frac{e}{\mu_{0}}{\left(\nabla s\right)}^{T}m$$

As reported in [16], the system of Equations (3)–(5) is solved in a self-consistent fashion by introducing boundary conditions for the electric potential u, the spin accumulation s and the electric current **j**_e_. For this purpose, Equation (3) is complemented by the source equation for the electric current **j**_e_ [16].

(6) $$\nabla \;\cdot \;j_{e}=0$$

By using this model, the electric potential u of a GMR element can be directly extracted from the simulation characterizing the GMR effect for an applied electric current. The respective material parameters of Equations (3)–(5) are specified in the following section.

### 2.2. Material Parameters and Dimensions

Due to their biocompatibility and chemical stability, iron-oxide nanoparticles made of magnetite (Fe_3_O_4_) are suitable for labels in biomedical applications [21,22,23,24,25,26,27]. Their synthesis is investigated in a wealth of literature, indicating that the superparamagnetic limit highly depends on the synthesis procedure [28,29,30].

In general, superparamagnetic properties can be observed below a particle diameter of 100 nm, where a magnetizing field **h**_zeeman_ of a few mT provokes a strong saturation magnetization M_s,p_ in the range of 20 emu/g to 90 emu/g [30,31]. In the simulation, the label’s saturation magnetization M_s,p_ is assumed to be 312 kA/m, which corresponds to 60 emu/g using a material density of 5.2 g/cm³ [32]. Following [30,31], a magnetizing z-field $\mu_{0}h_{\text{zeeman}}$ of 100 mT is applied, and the label’s used exchange constant A_ex,p_ of 10 pJ/m is extracted from [33].

**Figure 2:**
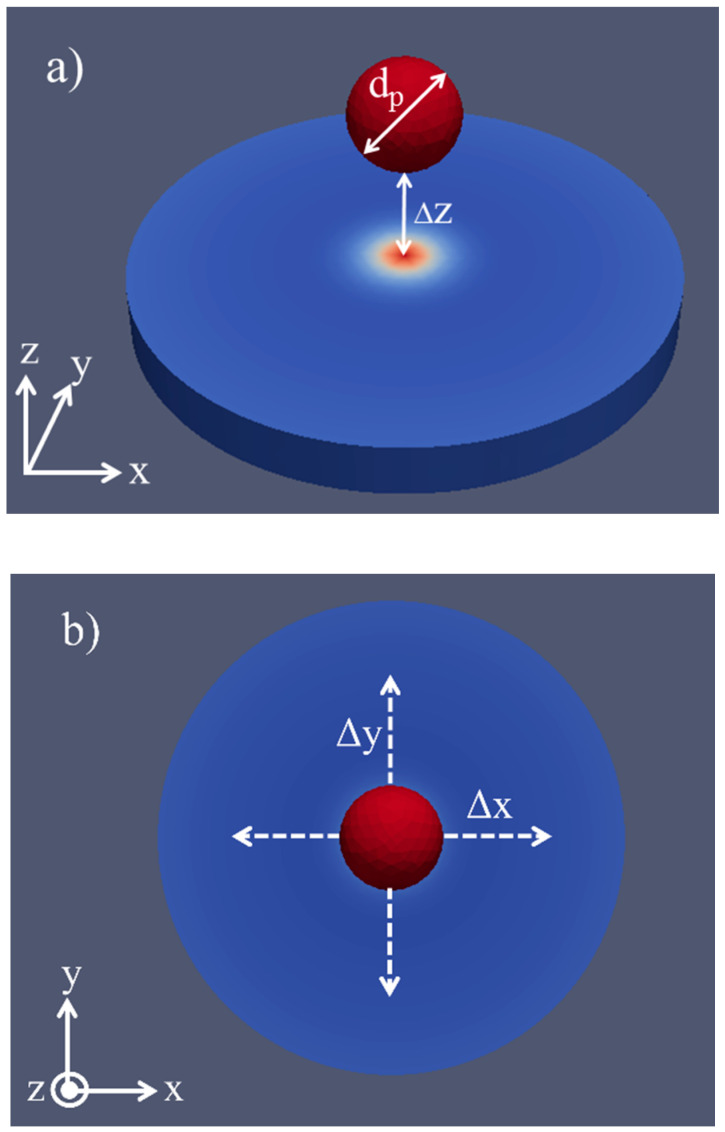
Simulation setup: (**a**) Variation of the label’s height and diameter. (**b**) Definition of the label’s shift in the x/y plane starting from the center of the free layer.

Both the diameter of the label (d_p_) as well as its lateral and vertical position relative to the sensor’s free layer are varied. While d_p_ receives values between 20 nm and 100 nm, the vertical position Δz is varied between 10 nm and 100 nm (Figure 2a). The horizontal and lateral position is defined as the label shift in the x- or y-direction (Δx/Δy) starting from the center of the circular free layer (Figure 2b). It is changed in the negative and positive x- and y-direction from 25 nm to 50 nm and 75 nm.

According to [34], the detection of a single magnetized label is possible as long as the size of the sensor structure is similar to the size of the label. Therefore, a sensor with a diameter in the order of 100 nm is needed to detect the specified magnetite labels. Typically, the free layer of a GMR element consists of a soft ferromagnetic material [35]. The formation of vortices in such submicron layers was theoretically and experimentally investigated in [36,37,38,39], showing that the formation of a vortex state highly depends on the aspect ratio of the layer. Ensuring a stable vortex appearance, this was complemented by Equation (3), which connects the layer thickness t_L_ to the layer diameter d_L_ and the exchange length of the material (l_ex,L_ = √2A_ex,L_/µ_0_M_s,L_²) [40].

(7) $$t_{L}\;>\;30\pi \frac{l_{\mathrm{ex},L}^{2}}{d_{L}}$$

**Figure 3:**
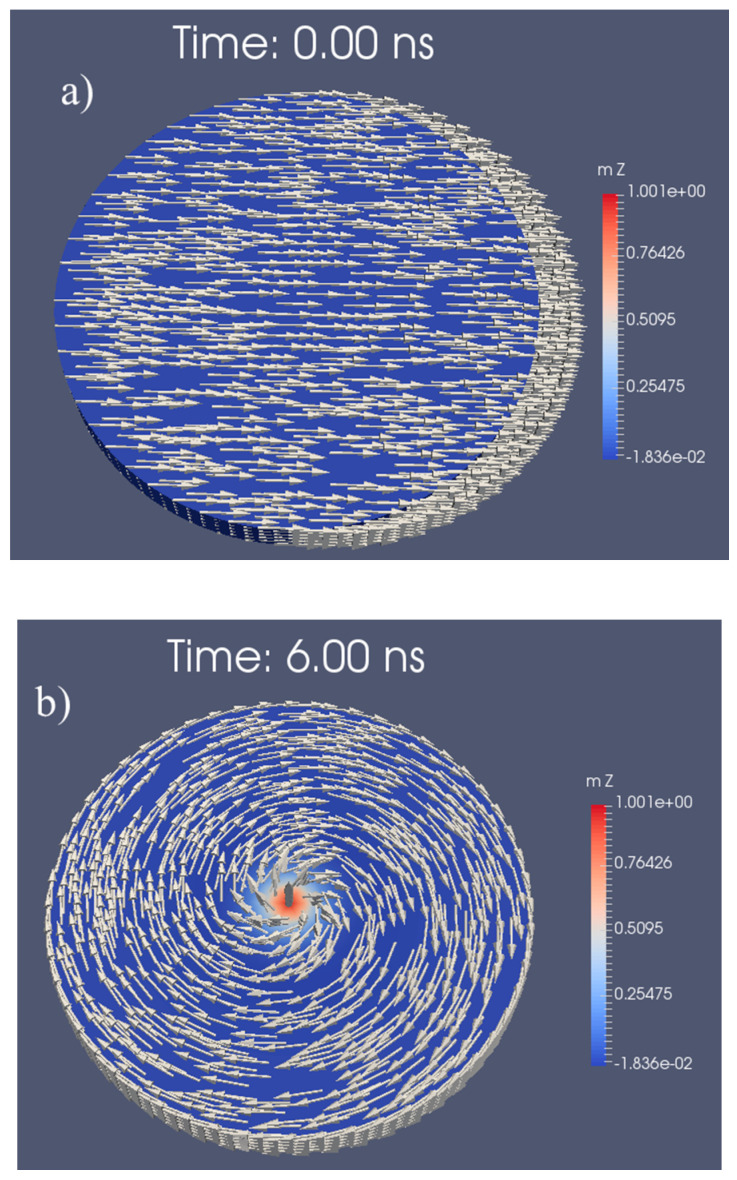
Vortex formation of a circular CoFe disk (d_L_= 100 nm, t_L_ =10 nm) starting (**a**) from the saturated state in the x-direction and (**b**) after a simulation time of 6 ns (**b**).

Using a CoFe alloy (M_s,L_ = 2.0 T [40], A_ex,L_ = 15 pJ/m [10]) as the free layer material, an exchange length l_ex_ of 3.1 nm is obtained. With the objective of d_L_ ≈ 100 nm, t_L_ has to be > 8.5 nm, and a layer thickness of t_L_ = 10 nm will lead to a stable vortex structure. This is confirmed by the simulation result displayed in Figure 3, where the vortex state occurs as the CoFe layers ground state after 6 ns.

Considering the exchange lengths of the label and the free layer (l_ex,p_ = 12.8 nm and l_ex,L_ = 3.1 nm), a tetrahedron mesh is generated using an element size of 3 nm. The damping constants α_L_ and α_p_ are set to 1.0, since only the steady state of the free layer’s magnetization will be used to compute the GMR effect within the self-consistent spin-diffusion model.

The single GMR element consists of the CoFe free layer [41], a 2 nm thick Cu layer and a 5 nm thick CoFeB fixed layer [35,42]. In order to satisfy Equations (5)–(7), further material parameters are required, namely, the diffusion constant D_0_, the conductivity C_0_, the spin-flip relaxation time τ_sf_, the coupling strength J and the polarization parameters β and β’ [43]. Except C_0_, these material parameters are taken from [16], while the conductivities of CoFe, CoFeB and Cu are assumed to be 2.5 MA/Vm [44], 0.3 MA/Vm [45] and 30 MA/Vm [46], respectively. Lastly, the fixed layer’s magnetization is pinned in the x-direction and an electric current of I = 8 mA is applied in the z-direction.

## 3. Results

### 3.1. Change in the Free Layer’s Magnetization

**Figure 4:**
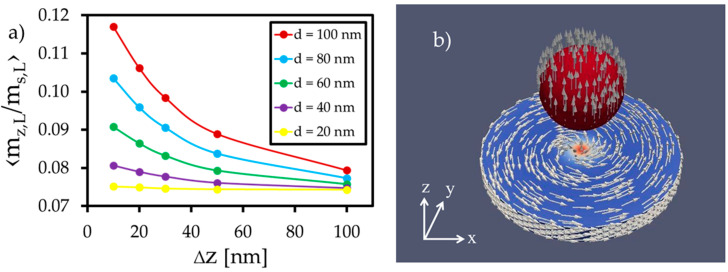
(**a**) Averaged z-component of the free layer magnetization during the simulation of different particle diameters placed in different heights in the center above the free layer. (**b**) Magnetization orientation of the magnetic vortex and the magnetized label after reaching the steady state of the simulation.

During the simulation, the magnetization direction of the label becomes parallel to the external z-field (Figure 4b). If the label is located in the center above the free layer, the effective stray field of the label acting on the free layer’s x/y plane will be balanced in all spatial directions, and the magnetic vortex will not move. Therefore, no significant magnetization change will occur in the x- or y-direction.

Due to the magnetizing external z-field, the z-component of the free layer increases compared to the setup without a label from m_z_/M_s,L_ = 0.01 to m_z_/M_s,L_ = 0.1. Additionally, the z-component is enhanced by the label’s stray field in the z-direction, which is strongest for large labels at short distances (Figure 4a).

**Figure 5:**
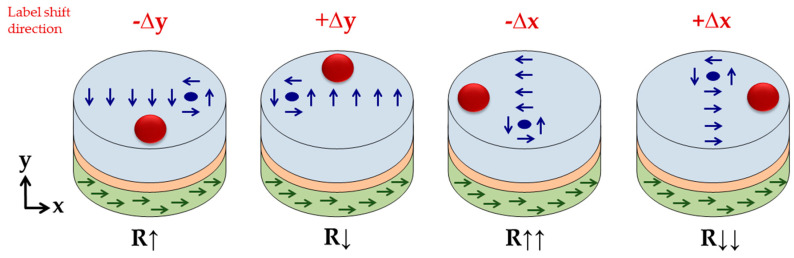
Sketch of the orthogonal movement of the magnetic vortex core with respect to the shift in the label. The shift in the magnetic vortex core leads to resistance changes across the GMR sensor stack, reaching its maximum resistance for label shifts in the x-direction.

By shifting the label in the x- or y-direction, the magnetization of the free layer changes significantly within the x/y plane. For example, an x-shift (∆x = 25 nm) of a label with a diameter of d_p_ = 80 nm placed in a height of ∆z = 20 nm leads to an increase in the relative averaged x-magnetization from 0.00 to 0.08. Accordingly, the rising spin alignment in the x-direction leads to an orthogonal movement of the vortex core, which slides in the positive y-direction, as illustrated in Figure 5. This can be observed vice versa for a label shift in the y-direction, since the magnetic stray field of the fixed layer is negligible due to the use of a synthetic antiferromagnet.

The orthogonal counterclockwise movement of the magnetic vortex core compared to the label’s shift direction is initiated by the spins surrounding the magnetic vortex core. At the edge of the core, the magnetization vector consists of magnetization components in the z- and x-/y-direction, depending on the respective magnetic domain. Without shifting the label, its stray field interacts with the out-of-plane vectors around the vortex core with perfect symmetry. Hence, the magnetic vortex remains in the center of the free layer.

**Figure 6:**
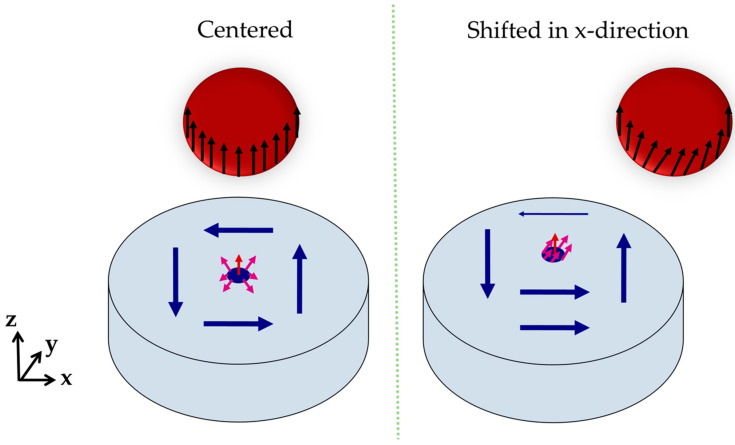
Schematic view on the vortex movement within the free layer by shifting the label in the x-direction. The labels shift urges the spins surrounding the vortex core to align more in the x-direction.

By shifting the label in the x-direction (Figure 6), the magnetization in the label center tilts in the x-direction. This is initiated by the stray field of the vortex and the pull-back mechanism of the z-component in the vortex center. The resulting x-component of the label bottom side enhances the x-magnetization of the free layer surface, similar to the increase in the z-component in center position (see Figure 4). This enhancement naturally shifts the vortex core in the y-direction, increasing the free layer’s net magnetization in the x-direction.

**Figure 7:**
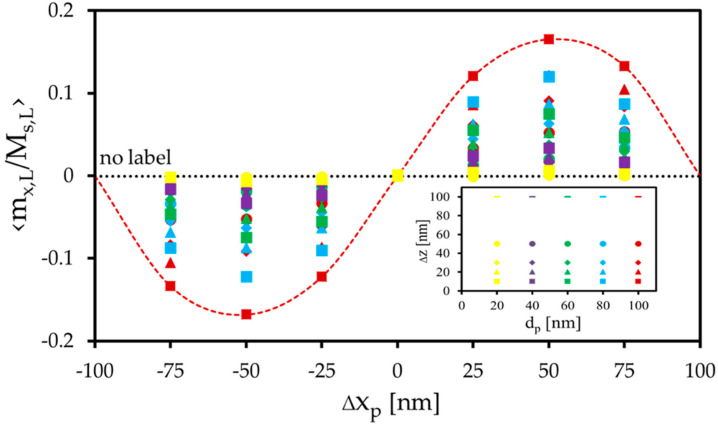
Change in averaged x-magnetization due to the x-shift in the magnetized label. The y-shift in the label leads to an equal distribution of the averaged y-magnetization. The inset shows the label diameters d_p_ and heights ∆z used for the simulation.

The amount of the magnetization change within the free layer can be manipulated by changing the diameter of the label and its distance to the layer surface, which is shown in Figure 7. Large particles and short distances generate relative magnetization changes up to 0.16 in the x- and y-direction, respectively.

It is noticed that the local magnetization change for a certain particle diameter d_p_ depends on the label’s shift length and the direction of the shift. This transfer curve shows properties of a sinusoidal function (Figure 7), where a label shift of 0.5d_L_ (50 nm) provokes the highest interaction. At this position, the label’s stray field urges the spins strongly to an alignment in the direction of the label’s shift. Towards the center, this force is reduced, and the label’s stray field acts more and more in all spatial directions on the free layer.

At the edges, this force is also reduced since the label slowly disappears from a position above the free layer’s surface. Thus, the magnetization change decreases towards the edges.

### 3.2. Resistance Changes due to the Label’s Stray Field

**Figure 8:**
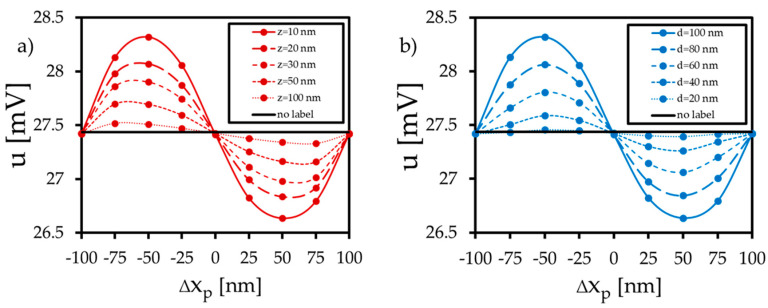
Simulated electric potential u of the GMR element due to the x-shift in the magnetized label. (**a**) Electric potential due to different label heights ∆z (d = 100 nm). (**b**) Electric potential due to different label diameters d (∆z = 10 nm).

**Figure 9:**
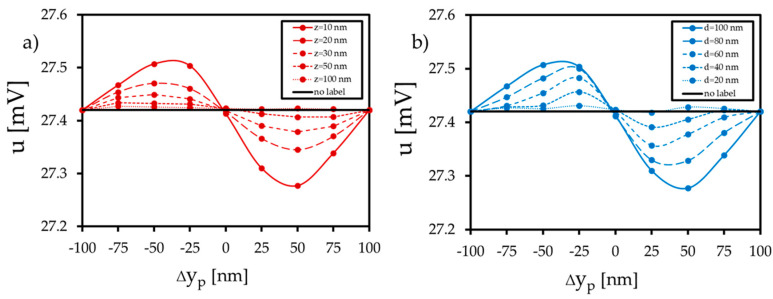
Simulated electric potential u of the GMR element due to the y-shift in the magnetized label. (**a**) Electric potential due to different label heights ∆z (d = 100 nm). (**b**) Electric potential due to different label diameters d (∆z = 10 nm).

In this section, the GMR from the magnetization patterns in the presence of the label is calculated following the procedure outlined in Section 2.2. Figure 8 and Figure 9 show the results of the potential change for the respective label shifts. According to the findings of the magnetic simulations, a label shift in the ±x-direction leads to a shift in the vortex core in the ±y-direction. Due to more parallel or antiparallel spin alignment between the fixed and the free layer, the electric potential is reduced or enhanced by 0.8 mV (at position 0.5d_L_) compared to the setup without or centered label. The amount of the potential change depends on particle size and distance (Figure 8). For the displacement in the ±y-direction (Figure 9), the distribution of the potential response is similar, but the amount of the potential change decreases significantly, as the fixed layer is aligned in the x-direction. At position 0.5d_L_, the electric potential is reduced or enhanced by 0.1 mV compared to the setup without a label. Due to the displacement in the ±y-direction, more spins align in the ±y-direction, and the vortex core slides in the ±x-direction. As a consequence, more spins align orthogonally to the pinning direction of the fixed layer, and the GMR is affected only weakly.

**Figure 10:**
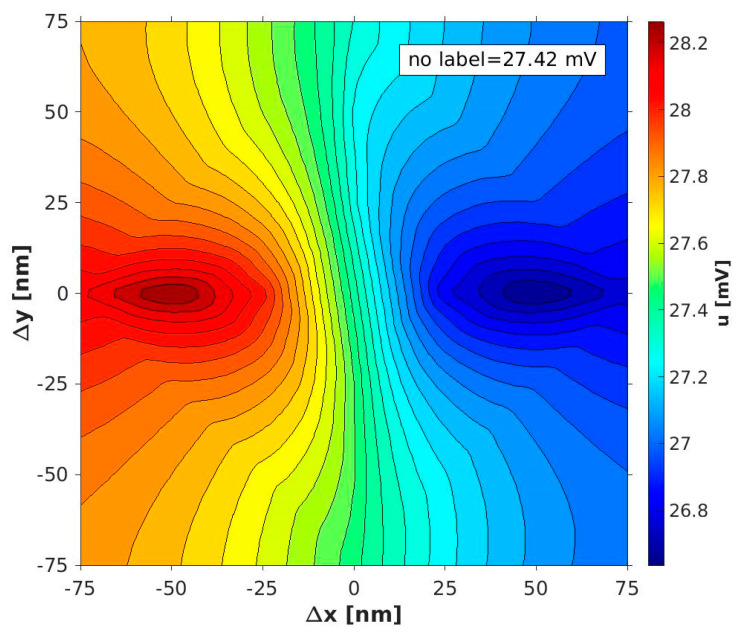
Interpolated electric potential within the x/y plane for a label with a diameter of d = 100 nm at a height of ∆z = 10 nm. The change in the electric potential reaches its maximum for label shifts parallel to the pinning of the fixed layer.

Without label displacement in the x- or y-direction, only the z-component of the free layer (and thus the magnetization of the small vortex core) is changed (Figure 4a). This magnetization change in a small region compared to the total surface of the free layer does not affect the GMR. This is confirmed by calculations and illustrated in Figure 10, where the center of the x/y plane shows an electric potential equal to the potential of the no label setup (u = 27.42 mV). Furthermore, it is obvious that the largest sensitivity is achieved by label shifts parallel to the magnetization of the fixed layer, in our case the x-coordinate. By using a sensor array with neighboring sensor elements, including orthogonally aligned fixed layers, similar sensitivity in the y-direction can be obtained.

## 4. Discussion

It is apparent that distinct GMR potential changes arise provided that the particle diameter is similar to the diameter of the sensor’s free layer. This was already theoretically reported in [34], where also a minimum distance of the label to the sensor surface of 0.2d_p_/2 was required. We met this criterion in the simulations as well, starting at a minimum height of 10 nm for the largest label with a diameter of 100 nm. Furthermore, it is pointed out that the x-displacement parallel to the fixed layer magnetization leads to larger potential changes compared to the displacement in the y-direction. Figure 8 shows a potential difference up to 0.8 mV between the setup without a label and the setup where the label is placed in the 0.5d_L_ position. This simulated change in the sensor’s response is within the range of analytical models and experimental work. As reported in [47], a resistance change of 20 mΩ has been generated by a spin-valve stripe detecting a single magnetite label with a diameter of 16 nm. In addition, [48,49] investigated the detection of multiple labels and showed that resistance and potential changes of 100 mΩ and 90 mV arise using GMR spin-valve stripes with a size of a few micrometers. According to [47], the transfer curve of the vortex GMR element (Figure 8) differs from the transfer curve of a GMR spin-valve stripe. For the circular vortex structure, the position of the highest potential drop or enhancement could be found at 0.5 d_L_ in the x-direction, whereas the center of the spin valve stripe was the position of highest interaction.

Noise is a limiting factor regarding the performance of the sensor system, since it blurs the sensor response. As reported in [10], the phase noise vanishes by the use of magnetic vortex structures. Therefore, only the thermal noise and the 1/f-noise contribute to the total noise of the GMR element [11]. Since the detection of a biomolecule is an application in the low frequency range, the influence of the 1/f noise becomes dominant, and the noise increases for small sensors [50]. For an estimate of the sensor noise in our case, the model reported in [11] can be consulted. Assuming a linear decay for the normalized Hooge parameter with an increasing aspect ratio of the free layer [11], the noise parameter S_v_ can be approximated to S_v_ ≈ 22 nV/√Hz using a frequency of f = 10 Hz. For this purpose, a connection of ten elements in series was assumed. Comparing this approximation with the results of [11] shows that S_v_ increases by a factor 4. The square root of the S_v_^2^ integration yields to a noise amplitude of u ≈ 104 nV, compared to an electric potential for the no label setup of u ≈ 27.4 mV, and a response just below 1 mV, which indicates an acceptable relation between the noise and the response of the simulated sensor structure.

## 5. Conclusions

In conclusion, we have used micromagnetic simulations to study the response of a GMR vortex structure to the stray field of a magnetized label.

If the label is centered above the vortex, only the average out-of-plane magnetization of the free layer will change from m_z_/M_s,L_ = 0.01 to m_z_/M_s,L_ = 0.1 depending on the label distance and the label diameter. For label shifts away from the sensor center, the orientation of the free layer’s average stray field is affected by the shift in the label. If the label that is magnetized in the z-direction will be moved in the x-direction, the average stray field produced by the label in the region of the free layer points in the x-direction. Hence, the x-directed magnetic domain in the vortex increases, which in turn leads to the movement of the vortex core in the y-direction. Based on this mechanism, the vortex core therefore always shifts in the orthogonal direction to the label shift.

In our specific setup, a parallel or antiparallel shift in the label along the pinning axis of the fixed layer, results in a modulation of the electric potential between 26.6 mV up to 28.3 mV. This depends on the distance and the diameter of the label, reaching a maximum for small distances (10 nm to 100 nm) and label diameters similar to the sensor diameter (100 nm).

The amount of the potential change is in agreement with already reported results of analytical modeling and experimental work [47,48,49], which was performed for spin valve stripes.

In summary, a submicron GMR element using a vortex free layer has the potential as a sensor for nanoscale label detection with direct electronic readout and low noise operation in lab-on-a-chip devices. Due to the basically hysteresis free operation of vortex sensors [7], we speculate that the detection of the labels can be done with higher reliability compared to the detection using standard elliptical GMR sensors.
